# Composition and nutritional role of gut microbiota on growth performance of pigs at different growth stages

**DOI:** 10.1017/S0007114525105989

**Published:** 2026-02-28

**Authors:** Kazuki Matsubara, Michi Yamada, Kazuhiro Hirayama

**Affiliations:** 1 Laboratory of Veterinary Public Health, Graduate School of Agricultural and Life Sciences, The University of Tokyo, 1-1-1 Yayoi, Bunkyo-ku, Tokyo 113-8657, Japan; 2 Department of Sustainable Agriculture, Rakuno Gakuen University, Ebetsu, 582 Bunkyodai Midorimachi, Ebetsu City, Hokkaido 069-8501, Japan; 3 Research Center for Food Safety, Graduate School of Agricultural and Life Sciences, The University of Tokyo, 1-1-1 Yayoi, Bunkyo-ku, Tokyo 113-8657, Japan

**Keywords:** Gut microbiota, Daily gain, SCFA, Free amino acids, Finishing pig, Growing pig

## Abstract

This study has investigated the relationship between the gut microbiota composition and the growth performance in pigs from birth to the finishing stage, focusing on nutrient metabolism. Of fifty-nine crossbred pigs [(Landrace × Large Yorkshire) × Duroc] from seven sows, individuals with high and low daily gain (DG) were assigned to high DG (HDG, *n* 11) and low DG (LDG, *n* 8) groups. Faecal samples collected at weaning (21 d), growing (95–106 d) and finishing (136–152 d) stages were analysed for amino acids, SCFA and microbial composition using 16S rRNA sequencing. Although birth and weaning weights were similar in both groups, the HDG group had significantly higher weights in the growing and finishing stages (*P* < 0·01). The microbial composition of the LDG group revealed a higher abundance of f_Lachnospiraceae;__ at weaning (*P* < 0·05), whereas the HDG group contained a higher abundance of g_Streptococcus and g_Prevotella 7 at the finishing stage (*P* < 0·05). Functional analysis revealed increased amino acid metabolism in the HDG group at the finishing stage (*P* < 0·05). During the growing stage, total free faecal amino acid content was low in the HDG group (*P* < 0·05); at weaning, levels of isobutyric and isovaleric acids, key amino acid fermentation products (*P* < 0·05, *P* < 0·01), were higher. These findings indicate growth stage-specific differences in the gut microbiota and metabolic profiles between groups with different growth performance, suggesting that microbial and metabolic characteristics may influence growth performance.

In pigs, growth performance – including average daily gain and feed conversion ratio – is influenced by various physiological and environmental factors. In recent years, the role of gut microbiota in shaping these traits has attracted attention. Several studies have reported associations between specific bacterial taxa and body weight (BW) or growth rates in pigs. For example, *Peptococcus* and *Eubacterium* have been linked to higher average daily gain, and *Treponema* and *Desulfovibrio* were found to have negative correlations^(1–3)^. In addition, a greater abundance of *Firmicutes* was identified in heavier piglets, whereas lighter individuals tend to carry more opportunistic bacteria^(4)^.

However, as most of these studies consider cross-sectional data from single time points, it is difficult to evaluate how differences in microbiota and microbial activity relate to growth performance throughout the production period. Recent work has described dynamic shifts in the composition of the gut microbiota across growth stages^(5–7)^; however, few studies have explored how microbial and metabolic profiles differ over time between pigs with divergent growth outcomes.

In addition to the microbial composition, microbial metabolites, such as SCFA and faecal amino acids, reflect microbial activity and host nutrient utilisation. SCFA provide energy and modulate gut health; conversely, amino acid levels in faeces may indicate protein digestibility or microbial proteolysis^(8,9)^. Despite their relevance, these functional indicators are rarely examined in studies of gut microbiota and growth performance.

In the present study, based on DG from birth to the finishing stage, we classified pigs as high daily gain (DG) and low DG. We then compared microbial composition and metabolic profiles for these groups at three critical time points – weaning, growing and finishing – using faecal samples collected from the same individuals at different time points. By combining 16S rRNA gene sequencing with analyses of faecal SCFA and amino acids, we aimed to clarify how the microbial characteristics of each growth stage are associated with long-term growth performance.

## Materials and methods

### Animals and experimental design

The animal experiment was approved by the Institutional Animal Care and Use Committee of Rakuno Gakuen University (No. DH21B1).

In total, 66 LWD piglets (Landrace × Large Yorkshire × Duroc) from seven sows bred at the Rakuno Gakuen Field Education and Research Center (Hokkaido, Japan) were followed from birth to finishing. Piglets were kept with their sow until weaning, which occurred when the piglets were approximately 21 d old; subsequently, piglets from the same sow were grouped together and transferred to a pen. When the pigs reached approximately 30 kg BW, they were allocated into multiple finishing pens. Pigs were fed commercial diets (FEED ONE CO., LTD) appropriate to their developmental stage that met the National Research Council standards^(10)^, as follows: suckling (up to approximately 21 d of age), early weaning (up to approximately 30 d of age), late weaning (up to approximately 70 d of age), growing (up to approximately 120 d of age) and finishing (after 120 d of age). Individual pigs were weighed at birth (0 d old), at weaning (approximately 21 d old), in the growing stage (95–106 d old) and in the finishing stage (136–152 d old). The pigs were slaughtered when they reached approximately 115 kg BW (139–189 d old).

Faecal samples were collected from the same individuals at three time points: weaning and in the growing and finishing stages (*n* 66 × 3 time points). Using sterile swabs, samples of fresh faeces were collected directly from the pig’s rectum and immediately placed in anaerobic containers (AnaeroPouch-Anaero, Mitsubishi Gas Chemical). Samples were transported under refrigeration and stored at −80°C until analysis.

Of the sixty-six pigs examined, fifty-nine pigs that were slaughtered without apparent disease were considered the final population. DG from birth to finishing was calculated for all pigs. For subsequent analysis, pigs with a DG of ≥ +1 sd within each sow were assigned to the high daily gain group (HDG, *n* 11), and pigs with a DG of ≤ –1 sd to the low daily gain group (LDG, *n* 8). Group allocation was retrospective and non-randomised; blinding was not performed.

### Bacterial DNA extraction and purification

To profile the gut microbiota and identify bacterial taxa associated with growth efficiency, faecal samples collected at weaning, the growing stage, and the finishing stage were analysed using 16S rRNA gene sequencing. Bacterial DNA extraction was performed in accordance with a previously described method^(11)^. Briefly, approximately 0·2 g of faecal sample was suspended in 5 ml of PBS, which was filtered through a 100 μm cell strainer (Corning, NY, USA) and subsequently centrifuged (4℃, 9000 × *g*, 10 min) to produce a pellet. The pellet was suspended in 20 ml of PBS, and the centrifugation was repeated. The pellet was resuspended in 1 ml of TRIS-EDTA (TE) buffer (10 mM TRIS-HCl, 20 mM EDTA). After the addition of lysozyme (Merck) at a final concentration of 30 mg/ml, the mixture was incubated for 1 h at 37℃ with gentle shaking. After shaking, purified achromopeptidase (FUJIFILM Wako Pure Chemical Corporation) was added at a final concentration of 2000 units/ml, and the mixture was incubated for 1 h at 37℃ with gentle shaking. Furthermore, sodium dodecyl sulfate (final concentration of 1 mg/ml) and proteinase K (final concentration of 1 mg/ml) (FUJIFILM Wako Pure Chemical Corporation) were added to the suspension and incubated for 1 h at 55℃ with gentle shaking. DNA extraction was performed using phenol/chloroform/isoamyl alcohol (25:24:1, NIPPON GENE CO., LTD); the extracted DNA was subsequently precipitated by the addition of an equal volume of isopropanol and 3 M sodium acetate (final concentration of 0·3 M) and centrifuged (4℃, 9000 × *g*, 10 min) to remove the supernatant. The obtained pellet was washed with 75 % ethanol and completely dissolved in 100 μl of TE buffer on ice. The obtained DNA was treated with DNase-free RNase (*NIPPON GENE Co., LTD*) solution at a final concentration of 10 μg/ml, incubated for 1 h at 37℃ with gentle shaking, purified with a 20 % polyethylene glycol 6000/2·5 M NaCl solution (Hampton Research), washed thoroughly with 75 % ethanol and dissolved completely in 100 μl of TE buffer on ice.

### 16S rRNA gene amplicon sequencing

The bacterial 16S rRNA gene was amplified using two-step tailed PCR with the universal primers 27Fmod (5′-AGRGTTTGATYMTGGCTCAG-3′) and 338R (5′-TGCTGCCTCCCGTAGGAGT-3′), which are specific to the V1–V2 hypervariable region of the 16S rRNA gene^(12)^. The DNA concentration after PCR was 16·7 ng/μl (range 11·9–20·4 ng/μl), and the amplification proceeded without issues. The amplicons were purified using AMPure XP (BECKMAN COULTER) and quantified using a Synergy H1 instrument (Bio TekSA) and the QuantiFluor dsDNA System (Promega). The quality of the amplicon was assessed using the dsDNA 915 Reagent Kit (Agilent Technologies) and a Fragment Analyzer (Agilent Technologies). 16S rRNA gene sequencing was conducted by Bioengineering Lab. Co., Ltd utilising the Illumina MiSeq platform (Illumina) in conjunction with the MiSeq Reagent Kit v3 (Illumina). This sequencing process generated 2×300 paired-end reads, aligning with the established Illumina protocols.

### Bioinformatic analysis

The sequencing data were analysed using Quantitative Insight into Microbial Ecology 2 (QIIME2 version 2022.8)^(13)^. Only the demultiplexed (paired-end) sequences whose beginning reads obtained from amplicon sequencing data using the fastx_barcode_splitter tool in FASTX-Toolkit (ver. 0.0.14) (http://hannonlab.cshl.edu/fastx_toolkit/) matched exactly the primer sequences used were selected. Primer sequences were removed from the selected reads using fastx_trimmer in FASTX-Toolkit. Sequences with a quality value of less than 20 were then removed using sickle (ver. 1.33) (https://github.com/najoshi/sickle), and sequences shorter than 130 bases in length and their paired sequences were discarded. Reads were joined using the paired-end read joining script FLASH (ver. 1.2.11)^(14)^ with the following parameters: sequence length of 310 bp after joining, read joining length of 230 bp and a minimum overlap of ten bases. The obtained amplicon sequencing data were imported into the QIIME2 package and analysed using the DADA2 pipeline^(15)^ for quality control after removal of chimeric sequences. On average, 91·0 % of reads were retained after the quality control process (range 87·5 %–95·0 %). The samples were assigned to specific taxonomy information, from phylum to species, using the SILVA 138 database. Furthermore, unclassified taxonomy was identified using NCBI BLAST. To estimate species diversity within samples, the sequences were rarefied at 10 000 depths. To assess the diversity of the microbiota, *α*-diversity and *β*-diversity were estimated using the QIIME2 plugin based on amplicon sequence variants (ASV), Shannon indices and weighted UniFrac distance. Functional predictions for the microbiota were generated using PICRUSt2^(16)^ using the MetaCyc database version 27.0 (https://metacyc.org)^(17)^.

### Analysis of faecal free amino acids

Faecal free amino acids were analysed to assess protein utilisation and intestinal absorption efficiency, which are directly linked to growth performance. All measurements were conducted using wet faecal samples without moisture correction. Diarrhoea was not present in any of the faecal samples. Approximately 200 mg of faeces was mixed in a 5:1 ratio (weight per volume) with 100 mM sterile PBS, mixed thoroughly and centrifuged (4℃, 14 800 rpm, 10 min). Then, 200 μl of the supernatant was mixed thoroughly with acetonitrile (FUJIFILM Wako Pure Chemical Corporation) and centrifuged (4℃, 14 800 rpm, 10 min) to remove protein. The supernatant was filtered through a polyvinylidene fluoride 0·45 μm membrane (Merck Millipore, ). A 100 μl aliquot of the processed samples was diluted with 350 μl of 100 mM PBS. Amino acids were derivatised with 4-fluoro-7-nitro-2,1,3-benzoxadiazole (NBD-F, Tokyo Chemical Industry Co., Ltd); 50 μl of 50 mM NBD-F (dissolved in acetonitrile) was added, and the solution was heated at 60°C for 1 min and then cooled on ice. Then, 300 μl of 5 mM HCl was added, and 2 μl of the reaction mixture was injected into an HPLC system (Chromaster, Hitachi) connected to an octadecylsilyl column (CAPCELL PAK C18 MGII S-5, 3·0 mm I.D. × 250 mm, OSAKA SODA CO., LTD) and with 10 mmol/L citrate buffer with 75 mmol/l NaClO_4_/CH_3_CN (1:1) at a flow rate of 0·4 ml/min. Fluorescence detection of the NBD-amino acids was performed at 530 nm with excitation at 480 nm. Amino acid concentrations were calculated using external standard curves constructed from serial dilutions of amino acid standards (FUJIFILM Wako Pure Chemical Corporation).

### Analysis of faecal SCFA

Faecal SCFA were analysed as indicators of microbial fermentation activity and energy production relevant to pig growth. SCFA analysis was performed on wet faecal samples without moisture correction. Diarrhoea was not present in any of the faecal samples. Approximately 50 mg of faeces was mixed thoroughly in a 20:1 (vol/wt) ratio with 6 % HClO_4_ and centrifuged (4℃, 14 800 rpm, 10 min). The supernatant was centrifuged again under the same conditions and filtered through a 0·45 μm polyvinylidene fluoride membrane. Then, 50 μl of the processed samples was injected into an HPLC system connected to a size exclusion chromatography column (GL-C610H-S, 7·8 mm I.D. × 300 mm, Hitachi Chemical Co., Ltd) with 3 mM HClO_4_ as the mobile phase, at a flow rate of 0·5 ml/min. To detect SCFA, the absorbance of the reaction mixture was monitored at 440 nm after the addition of 0·1 mM bromothymol blue/15 mM Na_2_HPO_4_/2 mM NaOH (pH = 9·6). The concentrations of individual SCFA were determined using external standard curves constructed from standard solutions of succinate, lactate, formate, acetate, propionate, isobutyrate, butyrate, isovalerate and valerate (FUJIFILM Wako Pure Chemical Corporation).

### Statistical analysis

Statistical analyses of growth performance, gut microbial taxonomic composition, gut microbial functional pathway prediction, concentrations of faecal free amino acids and SCFA and *α*-diversity of gut microbiota were performed using Welch’s *t* test in JMP Pro 16 software (SAS Institute). The *β*-diversity of the gut microbiota among groups based on weighted UniFrac distance was compared using permutational ANOVA in the QIIME2 diversity plugin. Results are presented as the mean (sd), and *P*-values less than 0·05 were considered to indicate a statistically significant difference. To evaluate the adequacy of our sample size, a *post hoc* power analysis was performed. Given the observed effect size (mean difference = 20·49, sd = 12·08), a sample size of *n* 19 (HDG group: *n* 11, LDG group: *n* 8) and a significance level of 0·001, the analysis yielded a statistical power of 0·93.

## Results

In total, nineteen pigs (HDG: 11, LDG: 8) were included in the final analysis, with faecal samples collected at three stages (weaning, growing and finishing; fifty-seven samples in total).

### Growth performance

There were no significant differences in birth BW between groups (Table 1). BW tended to be heavier at the weaning stage in the HDG group than the LDG group (*P* = 0·09). In the HDG group, pigs were significantly heavier at the growing and finishing stages than in the LDG group (*P* < 0·001). There was no significant difference in DG from birth to the weaning stage, but DG from both the weaning stage to growing stage and the growing stage to finishing stage was significantly greater in the HDG group than in the LDG group (*P* < 0·001).

**Table 1.** Body weight and average daily gain by growth stages for each group

	HDG group (*n* 11)		LDG group (*n* 8)		*P*
	Mean	sd	Mean	sd	
Birth, BW, (kg)	1·4	0·4	1·2	0·4	0·28
Weaner, BW, (kg)	6·2	1·2	5·2	1·2	0·09
Grower, BW, kg	68·5	7·7	57·4	5·4	< 0·001
Finisher, BW, kg	111·2	10·5	90·7	7·5	< 0·001
Weaner, ADG, kg	0·23	0·05	0·19	0·06	0·14
Grower, ADG, kg	0·78	0·09	0·67	0·06	< 0·001
Finisher, ADG, kg	1·0	0·11	0·78	0·07	< 0·001
Total ADG, kg	0·78	0·06	0·63	0·03	< 0·001

HDG, high daily gain; LDG, low daily gain; BW, body weight; ADG, average daily gain.

The mean value (sd) of each group is shown.

### Gut microbiota composition (16S rRNA gene amplicon sequencing)

Gut microbiota profiles were obtained from all fifty-seven faecal samples using 16S rRNA gene sequencing.

#### *α*-diversity

**Figure 1.**
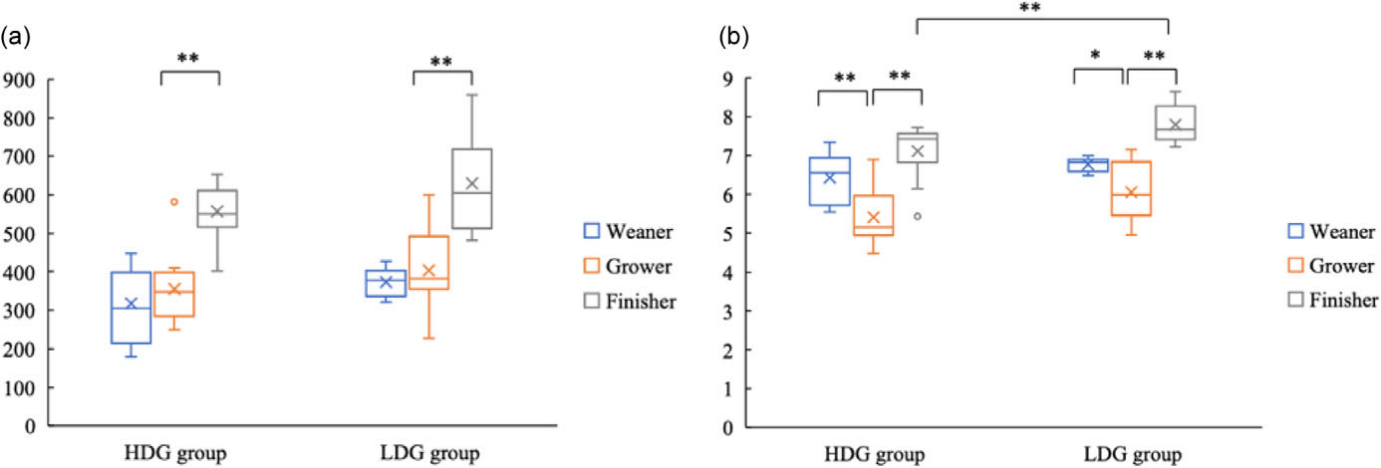
Measurement of *α*-diversity of gut microbiota of pigs at different growth stages (weaning stage: Weaner, growing stage: Grower, finishing stage: Finisher) between the high daily gain (HDG) group (*n* 11) and the low daily gain (LDG) group (*n* 8). (a) The box plots of amplicon sequence variants and (b) the box plots of Shannon’s index. *P < 0·05 and **P < 0·01, which are conducted by Welch’s *t* test.

Bacterial DNA was extracted from faeces collected at the weaning stage, the growing stage and the finishing stage of each group to examine differences in microbial composition. For the *α*-diversity, ASV did not differ significantly between groups at any stage. ASV were significantly higher at the finishing stage than the growing stage in both groups (*P* < 0·01) (Figure 1(a)). The Shannon index was significantly lower in the HDG group than in the LDG group at the finishing stage (*P* = 0·025). In both groups, the Shannon index was significantly lower at the growing stage than at the finishing stage (*P* < 0·01) and lower than at the weaning stage (the HDG group: *P* < 0·01; the LDG group: *P* = 0·033) (Figure 1(b)).

#### *β*-diversity

**Figure 2.**
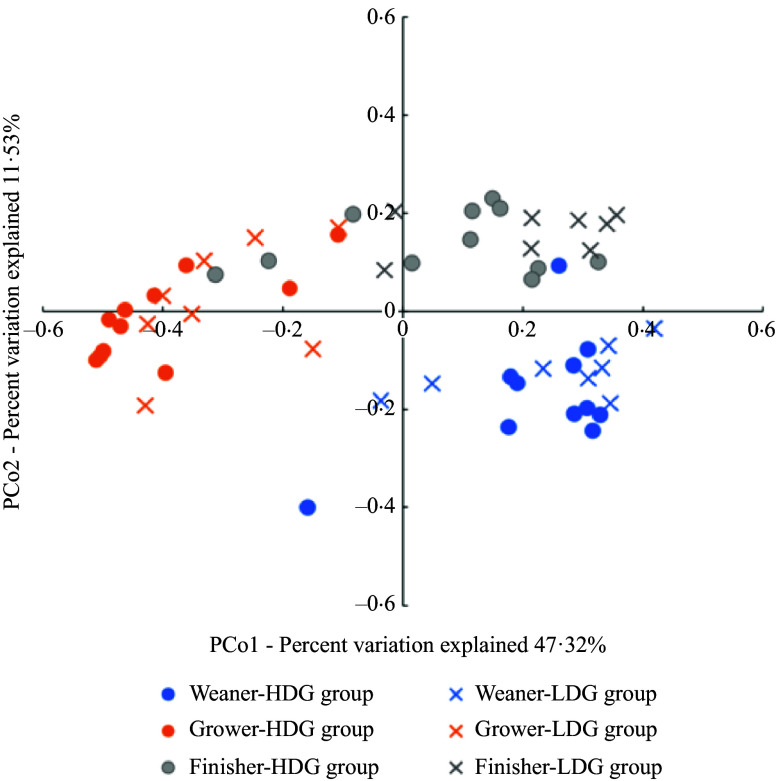
Measurement of *β*-diversity of gut microbiota of pigs at different growth stages (weaning stage: Weaner, growing stage: Grower, finishing stage: Finisher) between the high daily gain (HDG) group (*n* 11) and the low daily gain (LDG) group (*n* 8) by permutational ANOVA. Principal coordinates analysis plot based on the weighted UniFrac distances for gut microbiota composition in weaned piglets in each group at different growth stages.

The *β*-diversity was expressed as a principal coordinate analysis based on the weighted UniFrac distance. No significant differences between groups were found at any stage (Figure 2).

#### Differences in microbial taxa

**Figure 3.**
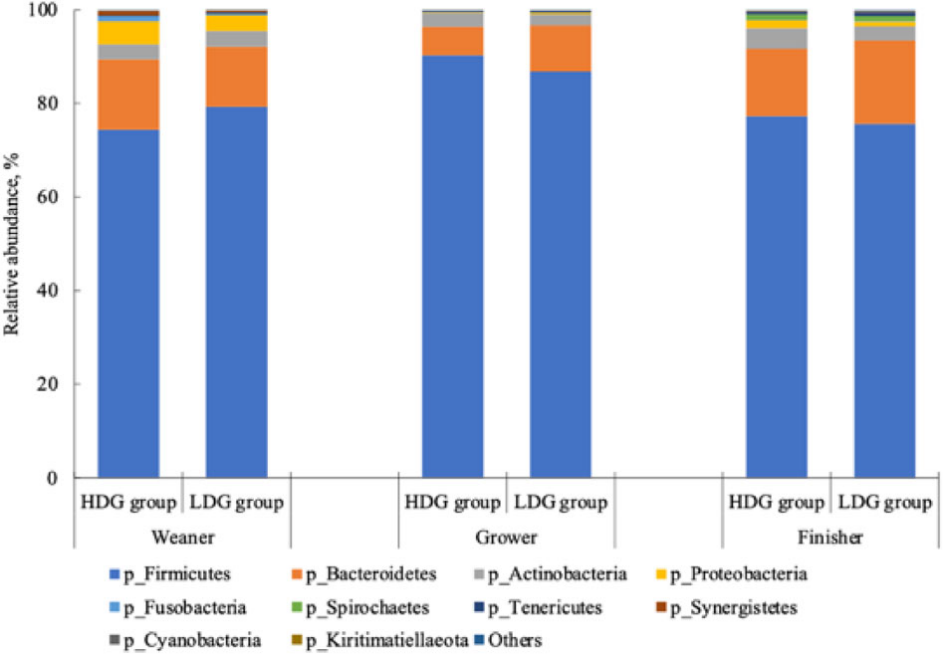
Relative abundance of predominant taxa detected in faeces of pigs at different growth stages (weaning stage: Weaner, growing stage: Grower, finishing stage: Finisher) between the high daily gain (HDG) group (*n* 11) and the low daily gain (LDG) group (*n* 8) at the phylum level.

**Figure 4.**
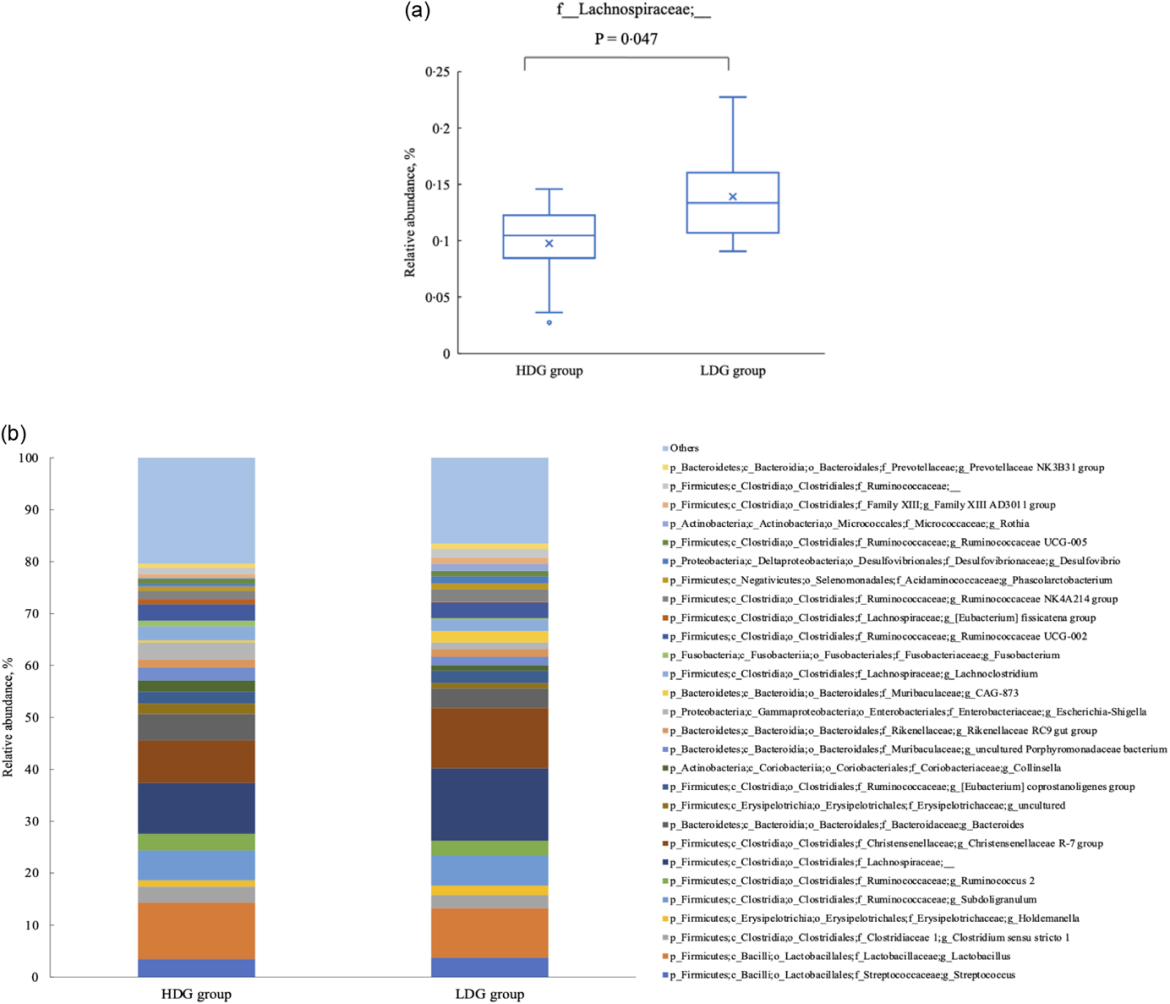
Composition of faecal microbiota at the weaning stage. (a) Relative abundance of f_Lachnospiraceae;__, between the high daily gain (HDG) group (*n* 11) and the low daily gain (LDG) group (*n* 8) at the weaning stage. (b) Predominant taxa by relative abundance detected in faeces of pigs at the weaning stage between the HDG group and LDG group.

**Figure 5.**
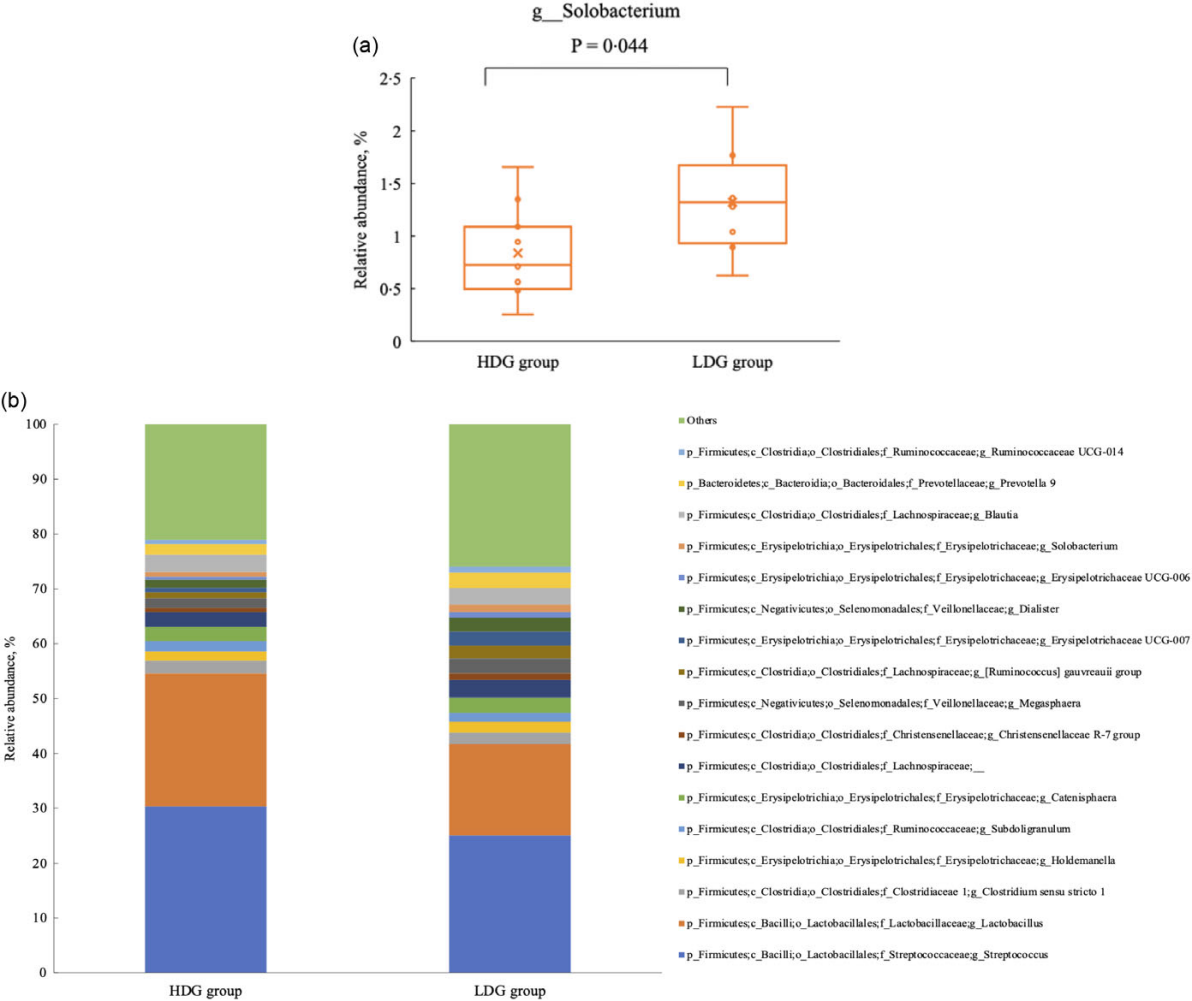
Composition of faecal microbiota at the growing stage. (a) Relative abundance of g_Solobacterium between the high daily gain (HDG) group (*n* 11) and the low daily gain (LDG) group (*n* 8) at the growing stage. (b) Predominant taxa by relative abundance detected in faeces of pigs at the growing stage between the HDG group and the LDG group.

**Figure 6.**
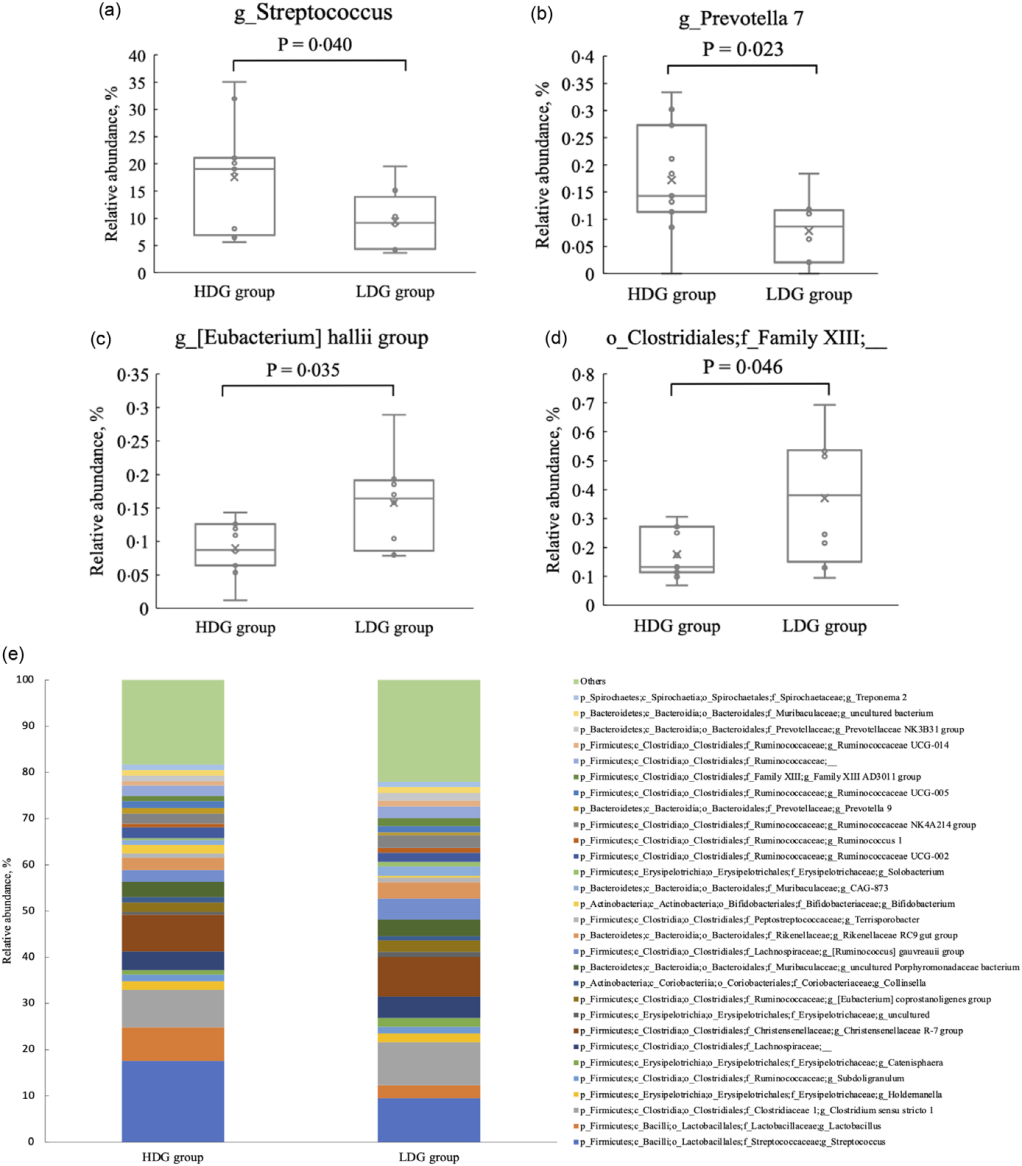
Composition of faecal microbiota at the finishing stage. (a) Relative abundance of g_Streptococcus between the high daily gain (HDG) group (*n* 11) and the low daily gain (LDG) group (*n* 8) at the growing stage. (b) Relative abundance of g_Prevotella 7 between the HDG group and the LDG group at the growing stage. (c) Relative abundance of g_[Eubacterium] hallii group between the HDG group and the LDG group at the growing stage. (d) Relative abundance of o_Clostridiales;f_Family XIII;__ between the HDG group and the LDG group at the growing stage. (e) Predominant taxa by relative abundance detected in faeces of pigs at the finishing stage between the HDG group and the LDG group.

There were no significant differences in the microbial composition between the groups at the phylum level. In both groups, *Firmicutes* had the highest relative abundance at the weaning stage, and the relative abundance of *Firmicutes*, *Bacteroidetes* and *Actinobacteria* (in this order) was higher at the growing and finishing stages (Figure 3). Bacteria with a mean relative abundance of at least 0·1 % or higher in each group were compared at the genus level. The relative abundance of f_Lachnospiraceae was significantly higher in the LDG group than in the HDG group at the weaning stage (*P* = 0·047) (Figure 4(a)). The five most abundant taxa at the weaning stage, in order of abundance, were g_Lactobacillus, f_Lachnospiraceae;__, g_Christensenellaceae R-7 group, g_Subdoligranulum and g_Bacteroides in the HDG group and f_Lachnospiraceae;__, g_Christensenellaceae R-7 group, g_Lactobacillus, g_Subdoligranulum and g_Bacteroides in the LDG group (Figure 4(b)). In the growing stage, the relative abundance of g_Solobacterium was significantly higher in the LDG group than in the HDG group (*P* = 0·044) (Figure 5(a)). The relative abundances of g_Streptococcus, g_Lactobacillus, g_Blautia, f_Lachnospiraceae;__ and g_Catenisphaera were highest in the HDG group, whereas the relative abundances of g_Streptococcus, g_ Lactobacillus, f_Lachnospiraceae;__, g_Blautia and g_Prevotella 9 were highest in the LDG group (Figure 5(b)). In the finishing stage, g_Streptococcus (*P* = 0·040) and g_Prevotella 7 (*P* = 0·020) were significantly more abundant in the HDG group than in the LDG group, and g_[Eubacterium] hallii group (*P* = 0·035) and o_Clostridiales;f_Family XIII;__ (*P* = 0·046) were significantly less abundant in the HDG group than in the LDG group (Figure 6(a)–(d)). The highest relative abundances of the bacteria were observed for g_Streptococcus, g_Clostridium sensu stricto 1, g_Christensenellaceae R-7 group, g_Lactobacillus and f_Lachnospiraceae;__ in the HDG group and g_Streptococcus, g_Clostridium sensu stricto 1, g_Christensenellaceae R-7 group, f_Lachnospiraceae;__ and g_[Ruminococcus] gauvreauii group in the LDG group (Figure 6(e)).

#### Functional pathway prediction using PICRUSt2

**Figure 7.**
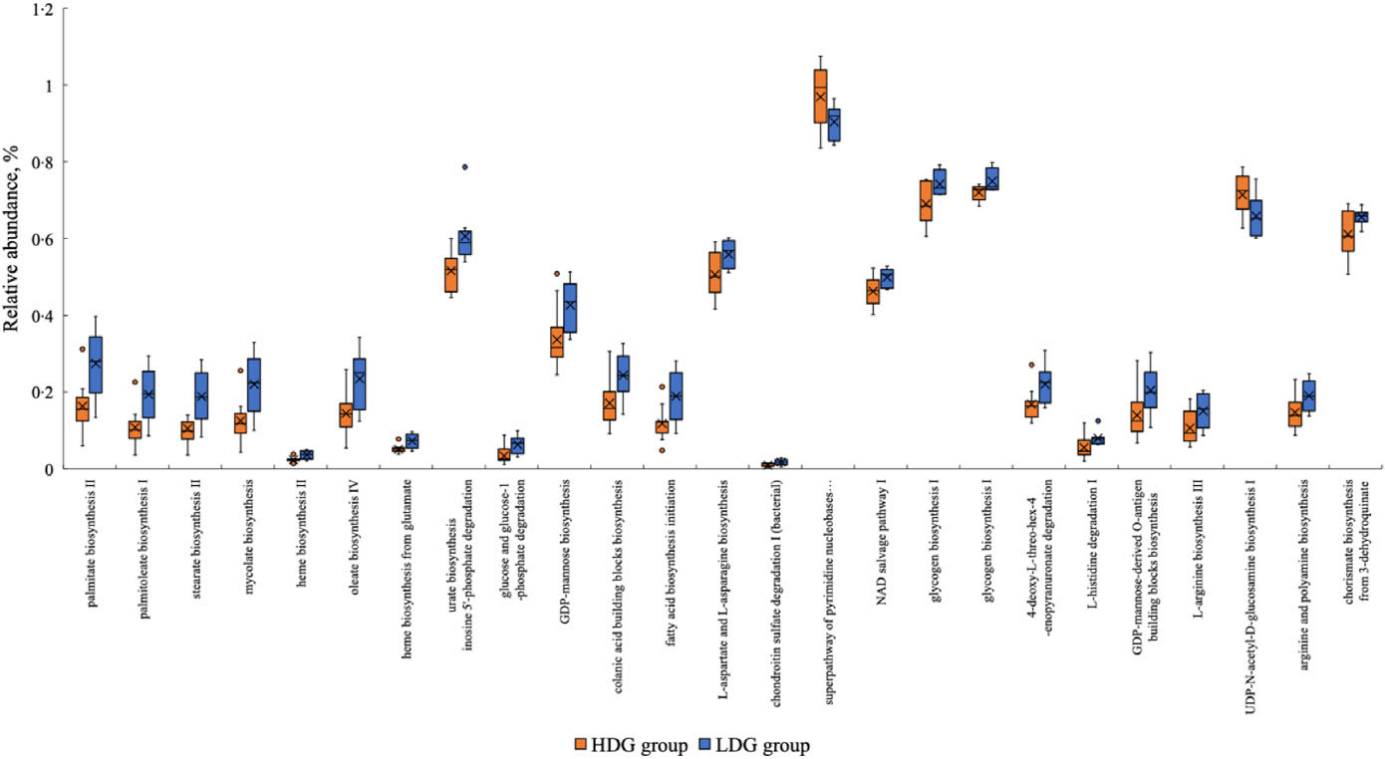
The metabolic pathways with the box plot using the MetaCyc database between the high daily gain (HDG) group (*n* 11) and the low daily gain (LDG) group (*n* 8) at the growing stage.

**Figure 8.**
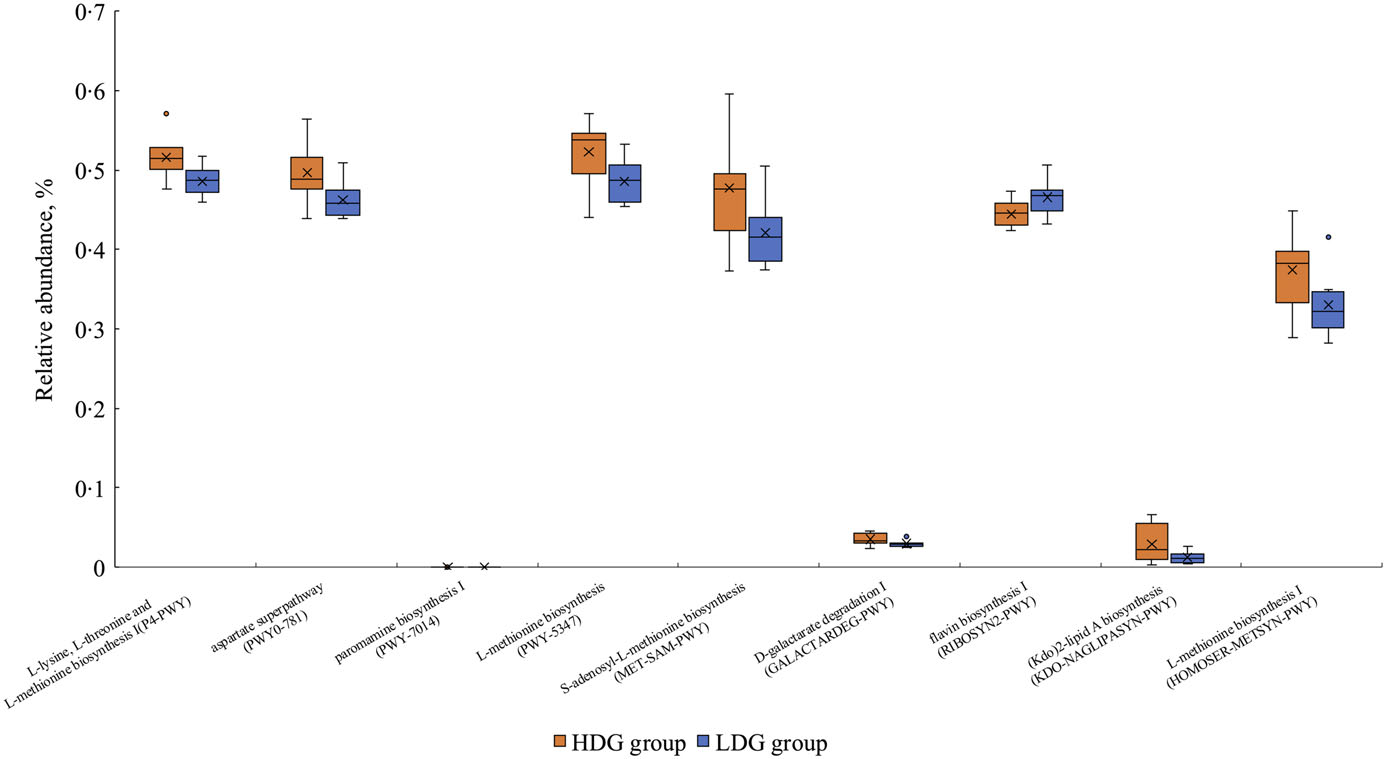
The metabolic pathways with the box plot using the MetaCyc database between the high daily gain (HDG) group (*n* 11) and the low daily gain (LDG) group (*n* 8) at the finishing stage.

PICRUSt2 was used to predict metagenomic functions in the microbial community from amplicon sequencing data obtained at weaning and the growing and finishing stages. The metabolic pathways that were significantly altered in each group based on the MetaCyc database are shown in Figure 7 for the growing stage (*P* < 0·05) and in Figure 8 for the finishing stage (*P* < 0·05). There were no significant differences at the weaning stage. In the growing stage, two pathways were significantly more abundant in the HDG group than in the LDG group: superpathway of pyrimidine nucleobases salvage and UDP-N-acetyl-d-glucosamine biosynthesis I. In contrast, twenty-two pathways were significantly more abundant in the LDG group than in the HDG group; these include long-chain fatty acid synthesis pathways such as palmitate biosynthesis II (bacteria and plants), palmitoleate biosynthesis I (from (5Z)-dodec-5-enoate) and stearate biosynthesis II (bacteria and plants) (Figure 7). In the finishing stage, eight pathways were significantly altered in the HDG group than in the LDG group; among these pathways, superpathway of l-lysine, l-threonine and l-methionine biosynthesis I, aspartate superpathway, superpathway of l-methionine biosynthesis (transsulfuration), superpathway of S-adenosyl-l-methionine biosynthesis and l-methionine biosynthesis I are involved in amino acid metabolism. Two pathways, paromamine biosynthesis I and flavin biosynthesis I (bacteria and plants), were significantly more abundant in the LDG group than in the HDG group (Figure 8). The metabolic pathways that were significantly different (*P* < 0·05) in relative abundance between the HDG and LDG groups during the growing and finishing stages are summarised in Table 2; the corresponding *P*-values are provided.

**Table 2.** *P*-values (*P* < 0·05) of functional pathway predictions in gut microbiota at the growing and finishing stages

Grower (HDG group: *n* 11, LDG group: *n* 8)	Higher	*P*
Palmitate biosynthesis II (bacteria and plants) (PWY-5971)	LDG	0·009
Palmitoleate biosynthesis I (from (5Z)-dodec-5-enoate) (PWY-6282)	LDG	0·011
Stearate biosynthesis II (bacteria and plants) (PWY-5989)	LDG	0·012
Mycolate biosynthesis (PWYG-321)	LDG	0·012
Heme biosynthesis II (anaerobic) (HEME-BIOSYNTHESIS-II)	LDG	0·013
Oleate biosynthesis IV (anaerobic) (PWY-7664)	LDG	0·014
Superpathway of heme biosynthesis from glutamate (PWY-5918)	LDG	0·014
Urate biosynthesis/inosine 5’-phosphate degradation (PWY-5695)	LDG	0·016
Glucose and glucose-1-phosphate degradation (GLUCOSE1PMETAB-PWY)	LDG	0·017
GDP-mannose biosynthesis (PWY-5659)	LDG	0·019
Colanic acid building blocks biosynthesis (COLANSYN-PWY)	LDG	0·020
Superpathway of fatty acid biosynthesis initiation (*E. coli*) (FASYN-INITIAL-PWY)	LDG	0·023
Superpathway of l-aspartate and l-asparagine biosynthesis (ASPASN-PWY)	LDG	0·024
Chondroitin sulfate degradation I (bacterial) (PWY-6572)	LDG	0·027
Superpathway of pyrimidine nucleobases salvage (PWY-7208)	HDG	0·031
NAD salvage pathway I (PYRIDNUCSAL-PWY)	LDG	0·031
Glycogen biosynthesis I (from ADP-d-Glucose) (GLYCOGENSYNTH-PWY)	LDG	0·032
4-deoxy-l-threo-hex-4-enopyranuronate degradation (PWY-6507)	LDG	0·032
l-Histidine degradation I (HISDEG-PWY)	LDG	0·037
Superpathway of GDP-mannose-derived O-antigen building blocks biosynthesis (PWY-7323)	LDG	0·038
l-Arginine biosynthesis III (via N-acetyl-l-citrulline) (PWY-5154)	LDG	0·041
UDP-N-acetyl-d-glucosamine biosynthesis I (UDPNAGSYN-PWY)	HDG	0·041
Superpathway of arginine and polyamine biosynthesis (ARG + POLYAMINE-SYN)	LDG	0·044
Chorismate biosynthesis from 3-dehydroquinate (PWY-6163)	LDG	0·048
Finisher (HDG group: *n* 11, LDG group: *n* 8)	Higher	*P*
Chorismate biosynthesis from 3-dehydroquinate (PWY-6163)	HDG	0·007
Aspartate superpathway (PWY0–781)	HDG	0·015
Paromamine biosynthesis I (PWY-7014)	LDG	0·021
Superpathway of l-methionine biosynthesis (transsulfuration) (PWY-5347)	HDG	0·022
Superpathway of S-adenosyl-l-methionine biosynthesis (MET-SAM-PWY)	HDG	0·035
d-Galactarate degradation I (GALACTARDEG-PWY)	HDG	0·036
Superpathway of d-glucarate and d-galactarate degradation (GLUCARGALACTSUPER-PWY)	HDG	0·036
Flavin biosynthesis I (bacteria and plants) (RIBOSYN2-PWY)	LDG	0·043
Superpathway of (Kdo)2-lipid A biosynthesis (KDO-NAGLIPASYN-PWY)	HDG	0·045
l-Methionine biosynthesis I (HOMOSER-METSYN-PWY)	HDG	0·046

HDG, high daily gain; LDG, low daily gain.

Only metabolic pathways with statistically significant differences (*P* < 0·05) between HDG and LDG groups are listed. ‘Higher’ indicates the group in which the predicted abundance of the pathway was significantly greater, as determined by PICRUSt2 analysis using the MetaCyc database.

### Concentration of faecal free amino acids

In the growing stage, the amounts of glutamic acid (*P* = 0·012), serine (*P* = 0·009), glycine (*P* = 0·017), threonine (*P* = 0·019), proline (*P* = 0·008), arginine (*P* = 0·016), valine (*P* = 0·023), methionine (*P* = 0·028), phenylalanine (*P* = 0·048), lysine (*P* = 0·011), tyrosine (*P* = 0·035) and total amino acids (*P* = 0·014) in the faeces were significantly higher in the LDG group than in the HDG group. Aspartic acid (*P* = 0·071), alanine (*P* = 0·070) and leucine (*P* = 0·074) tended to be higher in the LDG group than in the HDG group in the growing stage. In the finishing stage, serine (*P* = 0·084) tended to be higher in the LDG group compared with the HDG group (Table 3).

**Table 3.** Concentrations of faecal free amino acids by growth stages for each group (mol/g wet faeces)

	Weaner					Grower					Finisher				
	HDG group (*n* 11)		LDG group (*n* 8)		*P*	HDG group (*n* 11)		LDG group (*n* 8)		*P*	HDG group (*n* 11)		LDG group (*n* 8)		*P*
	Mean	sd	Mean	sd		Mean	sd	Mean	sd		Mean	sd	Mean	sd	
Asp	0·87	0·67	0·99	0·83	0·748	1·05	0·36	1·42	0·48	0·071	0·62	0·22	0·88	0·60	0·323
Glu	2·38	1·40	3·11	1·68	0·367	1·91	0·57	2·87	0·96	0·012	1·25	0·57	2·03	1·51	0·244
Ser	0·41	0·31	0·53	0·25	0·371	0·39	0·15	0·76	0·34	0·009	0·38	0·19	0·73	0·48	0·084
Gly	1·22	1·01	1·73	0·85	0·253	0·96	0·41	1·66	0·72	0·017	0·60	0·38	1·15	0·94	0·170
His	0·05	0·05	0·03	0·02	0·230	0·06	0·03	0·08	0·03	0·130	0·04	0·07	0·02	0·03	0·472
Thr	0·40	0·23	0·62	0·32	0·133	0·54	0·22	0·87	0·35	0·019	0·40	0·21	0·67	0·52	0·224
Ala	3·32	2·65	3·99	1·97	0·555	3·28	1·28	4·61	1·80	0·070	2·13	1·08	2·65	1·83	0·525
Pro	0·05	0·05	0·07	0·04	0·245	0·10	0·05	0·20	0·08	0·008	0·05	0·03	0·08	0·06	0·246
Arg	0·48	0·43	0·61	0·32	0·495	0·22	0·10	0·44	0·22	0·016	0·22	0·16	0·34	0·26	0·292
Val	1·06	0·76	1·32	0·78	0·503	0·75	0·43	1·27	0·51	0·023	0·65	0·29	1·05	0·85	0·292
Cys	0·04	0·08	0·01	0·01	0·226	0·03	0·01	0·02	0·02	0·158	0·04	0·04	0·03	0·03	0·441
Met	0·34	0·3	0·39	0·20	0·638	0·29	0·15	0·47	0·18	0·028	0·22	0·10	0·36	0·30	0·320
Leu	0·74	0·56	0·90	0·57	0·573	0·59	0·26	0·84	0·34	0·074	0·47	0·22	0·77	0·66	0·302
Ile	1·14	0·80	1·42	0·90	0·519	0·99	0·45	1·36	0·55	0·120	0·75	0·34	1·13	0·90	0·342
Phe	0·41	0·28	0·46	0·21	0·652	0·40	0·15	0·55	0·20	0·048	0·29	0·13	0·44	0·36	0·343
Lys	1·37	1·18	1·88	0·82	0·295	1·41	0· 62	2·64	1·22	0·011	1·05	0·56	1·99	1·54	0·164
Tyl	0·31	0·21	0·38	0·17	0·434	0·28	0·10	0·41	0·15	0·035	0·21	0·10	0·35	0·32	0·308
Total	14·6	10·3	18·5	9·0	0·415	13·2	4·5	20·5	7·4	0·014	9·40	4·3	14·7	10·9	0·269

HDG, high daily gain; LDG, low daily gain.

The mean value (sd) of each group is shown.

### Concentration of faecal SCFA

In the weaning stage, concentrations of isobutyric acid (*P* = 0·009) and isovaleric acid (*P* = 0·038) in the faeces were significantly higher in the HDG group than in the LDG group. In the growing stage, the concentration of valeric acid (*P* = 0·045) was significantly higher in the HDG group than in the LDG group. In the finishing stage, there were no significant differences in SCFA between the HDG and LDG groups (Table 4).

**Table 4.** Concentrations of faecal SCFA by growth stages for each group (mmol/g wet faeces)

	Weaner					Grower					Finisher				
	HDG group (*n* 11)		LDG group (*n* 8)		*P*	HDG group (*n* 11)		LDG group (*n* 8)		*P*	HDG group (*n* 11)		LDG group (*n* 8)		*P*
	Mean	sd	Mean	sd		Mean	sd	Mean	sd		Mean	sd	Mean	sd	
Succinate	0·36	0·45	0·11	0·20	0·121	1·16	1·26	0·85	0·78	0·520	0·13	0·23	0·11	0·10	0·735
Lactate	0·65	0·80	0·53	0·68	0·722	8·91	6·40	8·72	7·94	0·956	1·20	1·24	0·41	0·78	0·108
Formate	1·12	1·54	0·18	0·30	0·074	0·02	0·04	0·29	0·44	0·120	0·07	0·11	0·09	0·16	0·731
Acetate	27·0	7·78	21·9	7·40	0·167	46·0	7·20	49·0	11·9	0·544	37·9	8·94	34·9	8·29	0·464
Propionate	7·23	4·50	6·75	5·14	0·837	32·1	9·05	30·3	6·32	0·629	13·8	5·05	12·8	4·49	0·657
Isobutyrate	1·89	0·96	0·72	0·77	0·009	1·40	0·92	0·84	0·64	0·134	1·53	0·86	1·05	0·45	0·137
Butyrate	3·49	3·07	2·94	2·90	0·698	16·7	5·79	14·5	3·39	0·317	7·54	3·6	6·72	2·79	0·585
Isovalerate	6·29	3·91	3·27	1·69	0·038	3·33	2·11	2·60	2·12	0·465	5·25	2·46	3·86	1·97	0·188
Valerate	8·79	17·5	0·75	1·43	0·159	19·3	9·21	12·2	4·85	0·045	4·14	3·29	4·20	3·56	0·969
Total	56·9	27·2	37·2	13·7	0·056	128·9	19·3	119	18·7	0·291	71·6	20·4	64·2	13·2	0·351

HDG, high daily gain; LDG, low daily gain.

The mean value (sd) of each group is shown.

## Discussion

This study was performed to examine the relationship between growth performance, gut microbiota changes and nutrient metabolism in pigs. Among fifty-nine healthy pigs, individuals were classified into HDG and LDG groups based on their growth from birth to the finishing stage. The weaning weights in the HDG group tended to be higher than in the LDG group, which, consistent with previous finding, may have contributed to their subsequent growth performance^(18)^. In contrast, there were no clear differences in gut microbiota composition between the groups at weaning, suggesting the minimal contribution of gut microbiota to growth during weaning. However, the HDG group exhibited significantly higher DG during both the growing and finishing stages, and this was accompanied by notable differences in gut microbial composition and predicted functional pathways. Overall, these results suggest that post-weaning changes in the gut microbiota may be correlated with the final growth performance.

The *α*-diversity of pig gut microbiota increases during the pig’s transition from sow’s milk to plant-based diets, with the greatest diversity observed in the finishing stage^(19)^. Consistent with previous studies, ASV and the Shannon index significantly increased from the growing stage to finishing stage in both the HDG and LDG groups. A potential explanation for this is the decline in g_Streptococcus and g_Lactobacillus, which were abundant in earlier stages. Additionally, the Shannon index was significantly higher in the LDG group than the HDG group during the finishing stage. As the Shannon index considers both species richness and evenness, these results suggest that while ASV were similar for both groups, the microbiota in the HDG group was dominated by certain species, possibly reflecting the increased abundance of g_Streptococcus and g_Prevotella 7 observed in the HDG group at the finishing stage.

At all stages in this study, the gut microbiota was dominated by *Firmicutes*, *Bacteroidetes*, *Proteobacteria* and *Actinobacteria*. The relative abundance of *Proteobacteria* was particularly high at the weaning stage, consistent with previous reports^(20,21)^. However, there were no significant differences between groups at the phylum level. Specifically, at the taxonomic level, f_Lachnospiraceae was more abundant in the LDG group during weaning. While *Lachnospiraceae* is present in healthy piglets^(6)^, the abundance of *Lachnospiraceae* has been linked to post-weaning diarrhoea and is negatively correlated with DG^(22)^. Post-weaning diarrhoea, a major factor affecting piglet growth, was not observed in either group in the present study, suggesting that high abundance of f_Lachnospiraceae could not alone explain the DG differences. Previous studies have also found no substantial differences in gut microbiota composition between piglets with different BW at 21 d of age^(23)^. Collectively, these results suggest that, at least at weaning, the contribution of the gut microbiota to growth is limited compared with other factors, such as milk intake from the sow and birth weight.

In the growing stage, g_Solobacterium was significantly more abundant in the LDG group than in the HDG group. *Solobacterium* is a commensal bacterium that increases rapidly after weaning^(24)^, but its specific functions associated with growth performance in pigs remain unclear.

In the finishing stage, the abundance of g_Streptococcus and g_Prevotella 7 was significantly higher in the HDG group than in the LDG group. *Streptococcus*, a commensal bacterium linked to weight gain, is considered part of a healthy gut microbiota^(25,26)^, participating in the degradation of cellulose and lignin^(27)^. In this study, its ability to break down feed components may have improved growth performance. *Prevotella spp.*, which are known to increase after weaning, metabolise non-starch plant polysaccharides into SCFA^(19)^ and are associated with better feed efficiency and intake^(28)^. Higher *Prevotella spp.* levels in the finishing period likely contributed to higher feed intake and greater weight gain. Overall, these findings indicate that the gut microbial composition in the HDG group during the finishing stage may have been more functionally suited to efficient energy utilisation from feed; this could be related to the observed differences in growth performance. However, g_[Eubacterium] hallii group and o_Clostridiales;f_Family XIII;__ were significantly lower in the HDG group, but the impact of this difference remains unclear owing to a lack of relevant studies.

In the growing stage, concentrations of several free amino acids in faeces were significantly lower. Generally, higher faecal amino acid levels may result from increased microbial proteolysis or reduced digestion of host amino acid. In a study of Papua New Guinea highlanders, Tomitsuka et al. reported that faecal amino acid concentrations were higher in individuals with lower protein intake, likely because of enhanced microbial synthesis in the large intestine^(29)^. However, in this study, the metagenomic analysis revealed no difference in amino acid synthesis pathways between the LDG and HDG groups, suggesting differences in gut microbiota were not a key factor. There are a limited number of studies on faecal amino acid concentrations in pigs; however, Htoo et al. found that apparent ileal digestibility decreased with lower crude protein intake^(30)^. Although feed intake was not measured for individual pigs, a lower intake of crude protein in the LDG group may have led to a reduction in amino acid absorption efficiency and therefore higher faecal amino acid levels. At the weaning stage, isobutyric acid and isovaleric acid, which are fermentation products of amino acids, were significantly higher in the HDG group. Isobutyric acid, derived from valine deamination, is an indicator of dietary protein utilisation^(31)^. Higher ileal isobutyric acid levels are associated with lower levels of ‘residual feed intake’, indicating better feed efficiency^(1)^. These findings suggest that the pigs in the HDG group utilised proteins and amino acids more efficiently, which contributed to the greater weight gain. Collectively, these findings imply that, during the growing stage, differences in protein utilisation and amino acid absorption – rather than gut microbial composition – may be responsible for the different growth performance in the two groups.

In the analysis of the functional pathways of the microbiota, the activation of the long-chain fatty acid synthesis pathway was observed predominantly in the LDG group during the growing stage. The significance of long-chain fatty acids in swine faeces has not yet been determined; however, in experimental animals, it has been reported that elaidic acid, a type of *trans*-fatty acid, and palmitic acid, a type of SFA, downregulate the expression of tight junction genes and reduce intestinal barrier function, which may exacerbate metabolic diseases such as obesity and diabetes^(32)^. In pigs, weakened tight junctions have been shown to result in decreased feed intake, increased diarrhoea rate and delayed growth^(33)^. A similar mechanism may have contributed to the poor production performance of the LDG group in the present study.

In contrast, pathways related to amino acid metabolism were activated in the HDG group in the finishing stage. Although this study did not involve probiotic supplementation, the addition of probiotic *Lactobacillus plantarum* to feed was shown to increase the presence of *Prevotellaceae* and activate amino acid synthesis and metabolism^(34)^. In the present study, the relative abundance of g_Prevotella 7 was increased in the HDG group during the finishing stage. *Prevotella spp*. are known for their ability to ferment non-starch polysaccharides into SCFA and amino acid precursors, which can support improved feed efficiency and feed intake^(28)^. Therefore, it is plausible that the increased abundance of *Prevotella spp.* may lead to enhanced amino acid metabolism in the HDG group, which would support improved growth performance during the finishing stage.

### Conclusion

This study has identified stage-specific associations between the gut microbiota, nutrient metabolism and growth performance in pigs. At weaning, there were minimal differences in microbial composition between groups, suggesting the limited role of gut microbiota at this stage. During the growing stage, protein utilisation was more efficient in the HDG group, as indicated by lower concentrations of faecal free amino acids and a favourable metabolite profile. In contrast, in the LDG group, long-chain fatty acid synthesis pathways were activated, which may impair intestinal barrier function. In the finishing stage, the HDG group exhibited enhanced amino acid metabolism pathways, potentially supporting better feed efficiency and growth. In conclusion, the results of this study suggested that targeted interventions based on growth phase, relating to microbiota and nutrient optimisation, could improve swine production.
